# Supplementary figures and images for: Anabolic metabolism of autotoxic substance coumarins in plants
Source: PeerJ. 2023 Dec 6;11:e16508. doi: 10.7717/peerj.16508 (PMC10710134; doi:10.7717/peerj.16508)

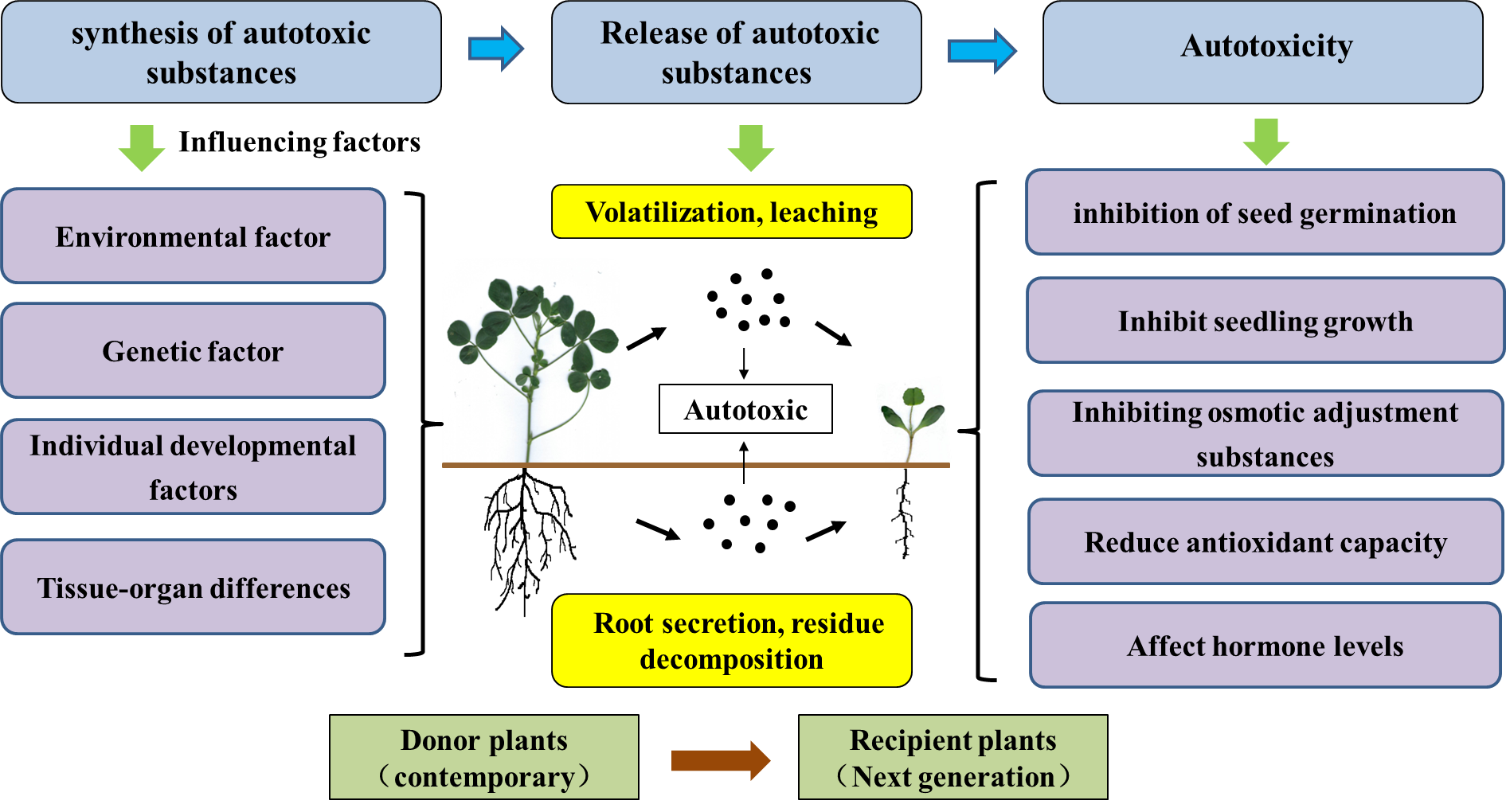

Supplement: Supplemental Information 1 — Three stages of autotoxicity (blue boxes): (1) the biosynthesis of autotoxic substances; (2) release of autotoxic substances; (3) autotoxicity. [file peerj-11-16508-s001.png]

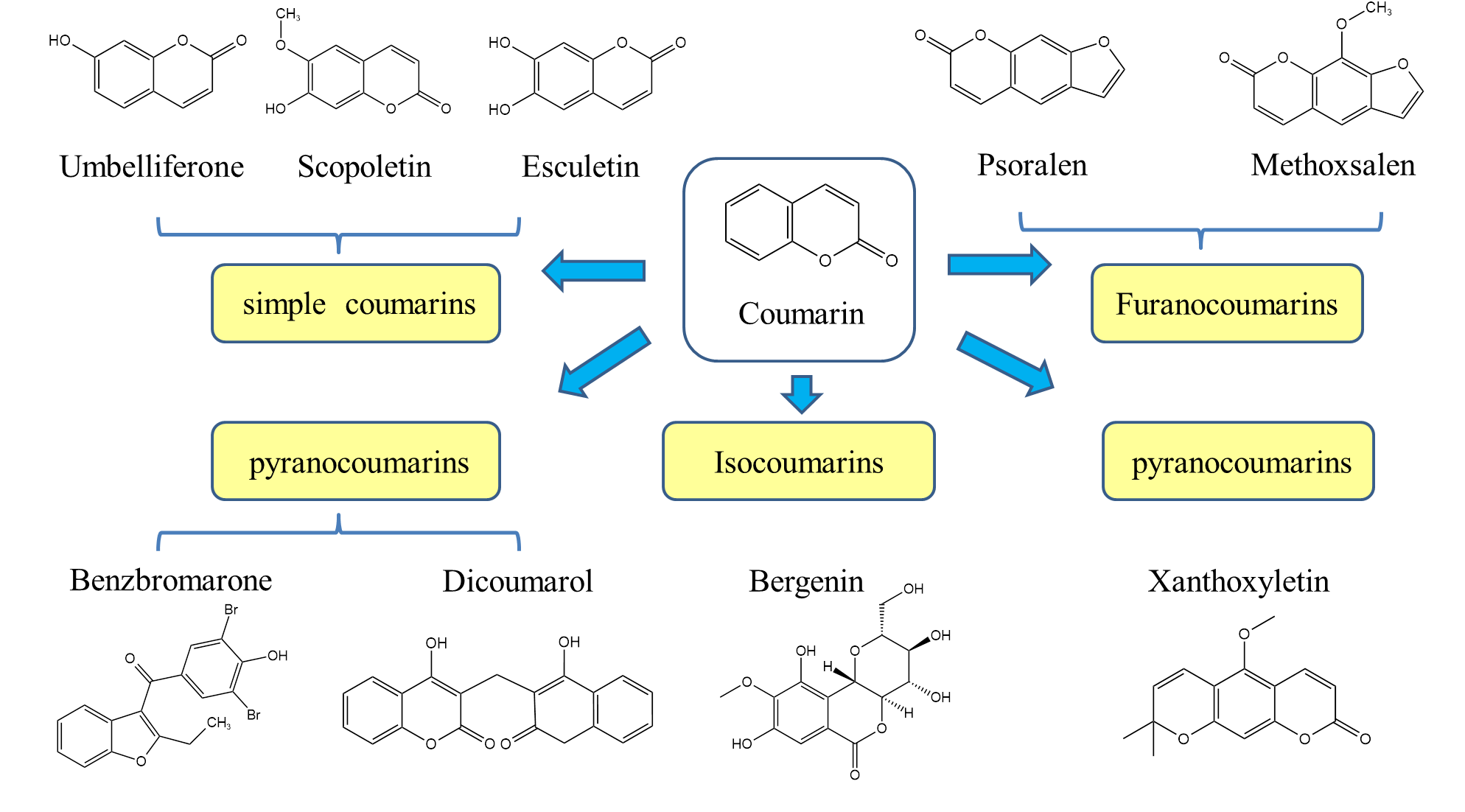

Supplement: Supplemental Information 2 — The yellow boxes are coumarins (Luo, 2017). [file peerj-11-16508-s002.png]

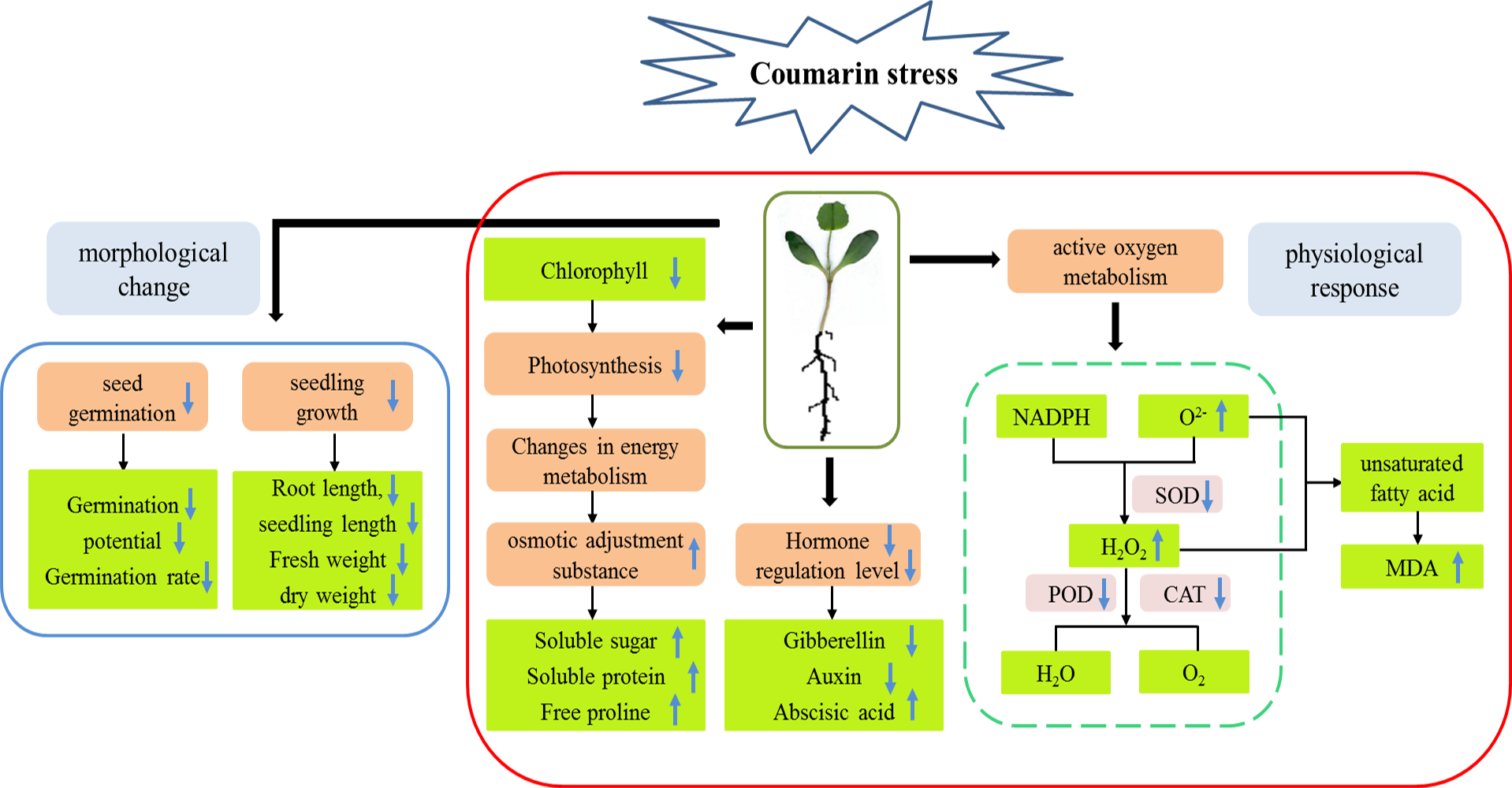

Supplement: Supplemental Information 3 — Effects of coumarins on plant phenotype (blue box), effects of coumarins on plant physiology (red box). The blue arrow up or down represents the increase or decrease of the index. [file peerj-11-16508-s003.png]

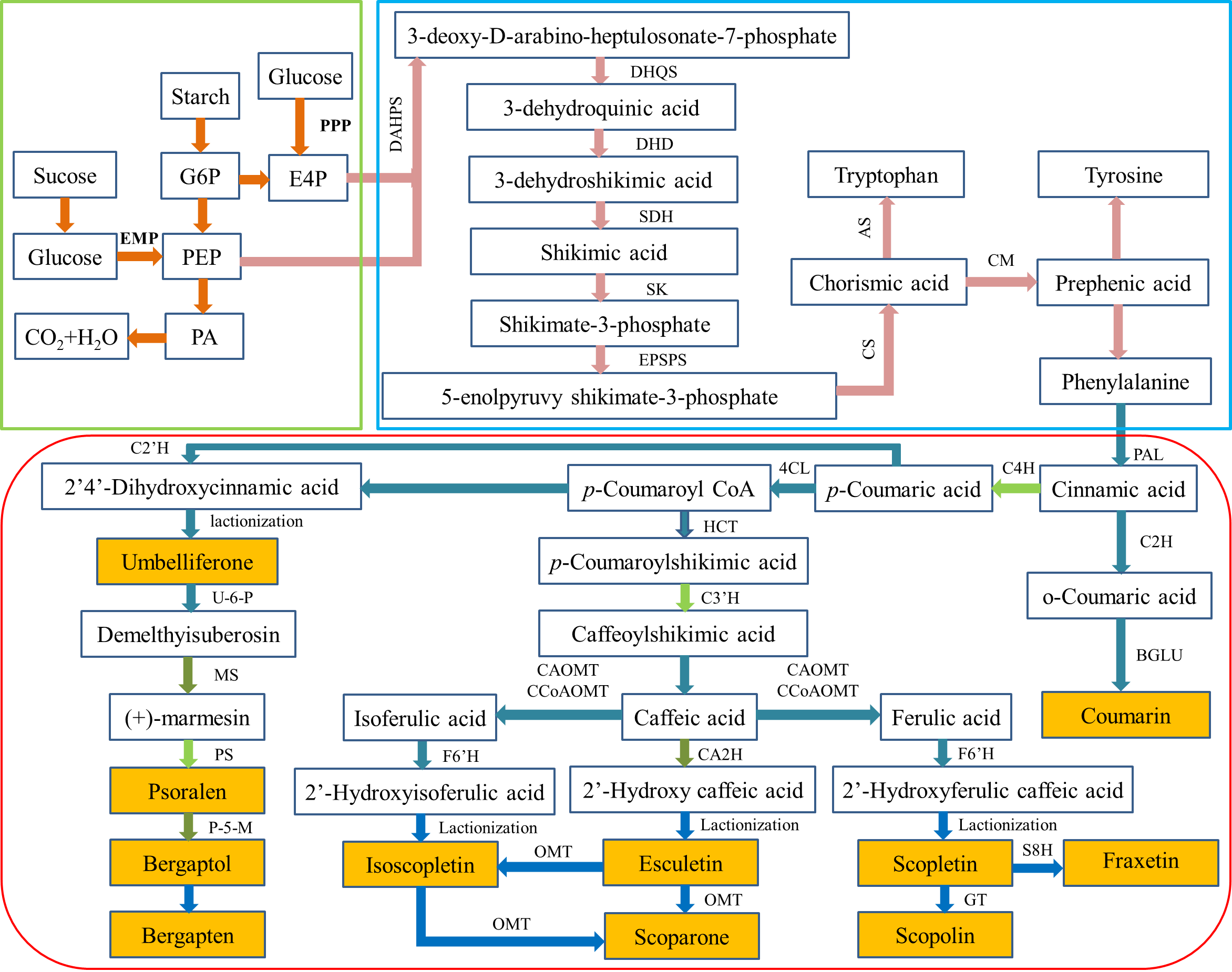

Supplement: Supplemental Information 4 — Primary metabolic process (green box), shikimic acid metabolic process (blue box) (Fu, Wei & Liang, 2021), phenylpropane metabolic process (red box). EMP: glycolysis; PPP: pentose phosphate pathway; DAHPS: 3-deoxy-D-arabino-heptulosonate-7-phosphate synthase; DHQS: 3-dehydroquinate synthase; DHD: 3-de-hydroquinic acid dehydrase; SDH: dhikimate dehydrogenase; SK: shikimate kinase; EPSPS: 5-enolppyruvylshikimate-3-phosphate synthe-tase; CS: chorismate synthase; AS: anthranilate synthetase; CM: chorismate mutase; PAL: phenylalanine ammonia lyase; C4H: cinnamic acid 4-hydroxylase; 4CL : 4-coumarate: coenzyme A ligase; HCT: hydroxycinnamoyl CoA shikimate; C3H: p-coumarate 3-hydroxylase; CAOMT: caffeic acid O-methyltransferase; CCoAOMT: caffeoyl-CoA O-methyltransferase; CA2H: caffeic acid 2-hydroxylase; F6’H: feruloyl-CoA 6′-hydroxylase; OMT: O-methyl-transferase; GT: glycosyltransferase; S8H: hydroxylation of scopoletin 8 hydroxylase; C2’H: Cinnamic acid 2′-hydroxylase; U-6-P: Umbelliferone 6-prenyltransferase; MS: Marmesin synthase; PS; Psoralen synthase; P-5-M: Psoralen 5-monoooxgenase; C2H: cinnamic acid 2-hydroxylase; BGLU: β-glucosidase (Zhao, Liu & Luo, 2015). [file peerj-11-16508-s004.png]

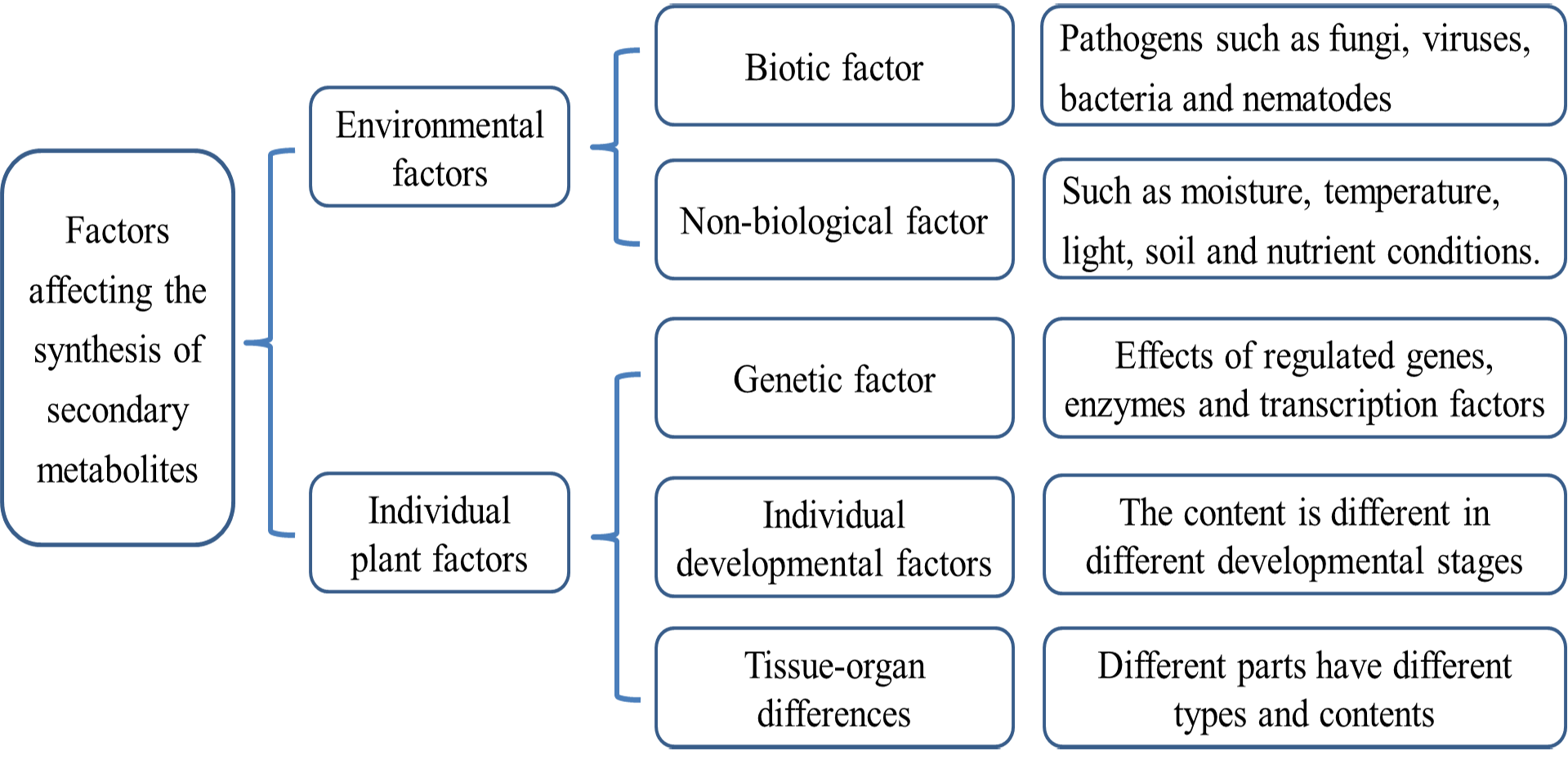

Supplement: Supplemental Information 5 — The factors affecting the content of coumarins in plants: environmental factors and individual plant factors (genetic factors, individual developmental factors and tissue-organ differences) (Verma & Shukla, 2015). [file peerj-11-16508-s005.png]
