# Supplementary material for: Anabolic metabolism of autotoxic substance coumarins in plants
Source: PeerJ. 2023 Dec 6;11:e16508. doi: 10.7717/peerj.16508 (PMC10710134; doi:10.7717/peerj.16508)
Supplement: Supplemental Information 6 [file peerj-11-16508-s006.docx]

**Table 1:**

**Types of plant autotoxic substances**

| Family | Plant species | Detection site | Types | Compounds | Reference |
| --- | --- | --- | --- | --- | --- |
| Gramineae | *Triticum aestivum* L*.* | Straw decomposition liquid | Phenolic acids | Benzoic acid, vanillic acid, eugenic acid, ferulic acid, cinnamic acid, etc. | Wang et al. (2018) |
|  |  |  | Coumarins | Coumarin |  |
|  | *Cenchrus spinifex* Cav. | Root, stem, leaf,  root exudates | Esters | Isobutyl octadecanol phthalate ester、Butyl undecyl phthalate ester | Wang et al. (2017) |
|  |  |  | Fatty acids | Linoleic acid, palmitic acid |  |
|  |  |  | Phenols | Phenol |  |
|  |  |  | Aldehydes | Isovanillin |  |
| Solanaceae | *Nicotiana tabacum* L. | Root exudates | Esters | Phthalic acid ester, alkyl esters, triethyl citrate, dibutylphthalate、dioctyl phthalate, diisocapryl phthalate | Deng et al. (2017) |
|  |  |  | Phenols | Dimethoxyphenol |  |
|  | *Cenchrus spinifex* Cav. | Rhizosphere soil | Esters | Di-n-hexyl phthalate, dibutylphthalate, dioctyl phthalate, diisocapryl phthalate | Ren et al. (2015) |
|  |  | Root exudates |  |  | Jiajun et al. (2017) |
| Umbelliferae | *Angelica sinensis* (Oliv.*)* Diels | Rhizosphere soil | Coumarins | Imperatorin | Xin et al. (2019) |
|  |  |  | Aldehydes | Vanillin |  |
| Araliaceae | *Panax pseudoginseng* Wall. var. *notoginseng (*Burkill*)* Hoo et Tseng | Root exudates,  rhizosphere soil | Phenolic acids | Benzoic acid, phthalic acid | Xiang (2016) |
|  |  |  | Fatty acids | Palmitic acid, stearic acid |  |
|  |  | Rhizosphere soil | Phenolic acids | Benzoic acid, ferulic acid, p-Hydroxybenzoic acid, vanillic acid, p-Coumaric acid | Wu et al. (2014) |
|  | *Panax quinquefolius* L. | Fibrous root residues | Phenolic acids | Syringic acid, p-Coumaric acid, ferulic acid | Li ＆ Jiang (2018) |
|  |  |  | Aldehydes | Vanillin |  |
| Scrophulariaceae | *Rehmannia glutinosa* Libosch. | Rhizosphere soil | Phenolic acids | p-Hydroxybenzoic acid, coumaric acid, syringic acid, 3,4-dihydroxybenzoic acid | Zhang (2015) |
|  |  |  | Aldehydes | Vanillin |  |
|  |  | Soil | Phenolic acids | Vanillic acid, alcohols, D-mannitol | Li et al. (2010) |
|  |  |  | Esters | 2[4'-hydroxyphenyl]-ethyl hexacosanoate |  |
|  |  |  | Sterols | Daucosterol, β-sitosterol |  |
| Labiatae | *Salvia miltiorrhiza* | Stem, leaf,  root decomposing liquid | Alcohols | 2-ethylhexanol | Wang (2021) |
|  |  |  | Alkanes | Undecane |  |
|  |  |  | Esters | Ethylhexyl benzoate |  |
| Ranunculaceae | *Paeonia ostii* T. | Rhizosphere soil | Phenols | Paeonol, ferulic acid, cinnamic acid, coumarin | Qin et al. (2009) |
|  |  |  | Aldehydes | Vanillin |  |
| Asparagaceae | *Asparagus officinalis* L. | Cultures on agar medium | Carboxylic acids | Oxalic acid, succinic acid, tartaric acid | Yeasmin et al. (2014) |
| Chenopodiaceae | *Beta vulgaris* L. | Water-soluble extract of residue | Phenolic acids | Hydroxybenzoic acid, p-coumaric acid, vanillic acid | Hegab et al. (2008) |
|  |  |  | Coumarins | Coumarin |  |
|  |  |  | Flavonoids | Protocatechuic acid |  |
| Liliaceae | *Lilium brownie* var. *viridulum* | Root exudates | Esters | 25 species | Ma (2019) |
|  |  |  | Phenolic acids | 6 species |  |
| Malvaceae | *Gossypium herbaceum* L. | Root, stem, root exudates | Phenolic acids | Ferulic acid, p-hydroxybenzoic acid, gallic acid, vanillic acid | Gui, Yan ＆ Jian (2013) |
| Cucurbitaceae | *Cucumis melo* L. | Root exudates | Phenolic acids | Gallic acid, phthalic acid, syringic acid, salicylic acid, ferulic acid, benzoic acid, cinnamic acid | Yang (2014) |
|  | *Citrullus lanatus* (Thunb.) Matsum. et Nakai | Root, stem, leaf,  root exudates | Phenolic acids | Benzoic acid, cinnamic acid, salicylic acid | Zheng et al. (2011) |
|  |  |  | Aldehydes | Vanillin |  |
|  |  |  | Esters | Dioctyl phthalate、diisobutyl phthalate |  |
| Vitaceae | *Vitis vinifera* L. | Root exudates | Alkanes | Hexamethyl-epoxytrisilane | Guo et al. (2012) |
|  |  |  | Fatty acids | Palmitic acid, citric acid, aconitic acid, gallic acid |  |
| Rosaceae | *Fragaria ananassa* Duch. | Root exudates | Fatty acids | Lactic acid, succinic acid | Kitazawa et al. (2005) |
|  |  |  | Phenolic acids | Benzoic acid, p-hydroxybenzoic acid |  |
|  |  |  | Carboxylic acids | Adipic acid |  |
| Compositae | *Mikania micrantha* Kunth | Root | Esters | Ethyl caffeate, ethyl ferulate | Xu et al. (2013) |
|  |  |  | Phenolic acids | 3,5-di-*O*-caffeoylquinic acid |  |
|  |  |  | Terpenoids | 9-isobutyryloxy-10hydroxythymol |  |
|  | *Chromolaena odoratum* | Overground part | Flavonoids | Chalcone, flavanone, flavonol | Lei (2011) |
| Leguminosae | *Vicia faba* L. | Root exudates | Phenolic acids | Benzoic acid, adipic acid, p-hydroxybenzoic acid, Salicylic acid, malonic acid, vanillic acid, | Asaduzzaman ＆ Asao (2012) |
|  |  |  | Fatty acids | Lactic acid, succinic acid, malic acid |  |
|  | *Vigna unguiculata* (Linn.) Walp. | Continuous cropping soil | Phenolic acids | 4-hydroxybenzoic acid, Phenylacetic acid, cinnamic acid, phthalic acid | Huang (2010) |
|  | *Arachis hypogaea* Linn. | Rhizosphere soil | Phenolic acids | Vanillic acid, p-hydroxybenzoic acid, coumaric acid | Huang et al. (2013) |
|  |  |  | Coumarins | Coumarin |  |
|  | *Medicago* *sativa* L. | Leaf | Phenolic acids | Salicylic acid, p-hydroxybenzoic acid | Ghimire et al. (2019) |
|  |  |  | Coumarins | Scopolamine, quercetin |  |
|  |  | Plant, rhizosphere soil extracts | Phenolic acids | 4-hydroxybenzoic acid, chlorogenic acid, caffeic acid, ferulic acid, coumarin, L-conglycine | Rong, Shi ＆ Sun (2016) |
