# Supplementary material for: Anabolic metabolism of autotoxic substance coumarins in plants
Source: PeerJ. 2023 Dec 6;11:e16508. doi: 10.7717/peerj.16508 (PMC10710134; doi:10.7717/peerj.16508)
Supplement: Supplemental Information 7 [file peerj-11-16508-s007.docx]

**Table 2:**

**Types of plant coumarins**

| Family | Plant species | Detection site | Coumarin type | compounds | Reference |
| --- | --- | --- | --- | --- | --- |
| Thymelaeaceae | *Stellera chamaejasme* L. | Root | Simple coumarins | Scopoletin | Liang (2005) |
|  |  |  | Bicoumarins | Daphnoretin |  |
|  |  | Flower | Simple coumarins | 7-hydroxy-8-methoxycomarin, 5,7-dimethoxycoumarin,  scopoletin, Umbelliferone, | Zhou et al. (2021) |
|  |  |  | Bicoumarins | Daphnetin, edgeworthin, daphnoretin |  |
| Umbelliferae | *Angelica dahurica* (Fisch. ex Hoffm.) Benth. et Hook. f. ex Fanch. et Sav. | Decoction | Furanocoumarins | Imperatorin, isoimperatorin, oxypeucedan | Zhang (2012) |
|  | *Glehnia littoralis* Fr. Schmidt ex Miq. | Decoction | Furanocoumarins | Bergapten, xanthotol |  |
| Convolvulaceae | *Ipomoea cairica* (L.) Sweet | Leaf, stem | Simple coumarins | Umbelliferone, Scopoletin | You et al. (2014) |
| Rutaceae | *Citrus maxima* (Burm) Merr. | Grapefruit skin | Simple coumarins | 7-methylcoumarin, 7-methoxycoumarin,  7-ethoxy-4-methylcoumarin | Hao (2019) |
|  |  |  | Bicoumarins | Acenocoumarol |  |
| Leguminosae | *Melilotus officinalis* (L.) Pall. | Leaf, stem, flower | Simple coumarins | Coumarin, Umbelliferone, Scopoletin,  7-Methylcoumarin, 4-methoxycoumarin,  3-hydroxycoumarin, dihydrocoumarin,  scoparone | Tang ＆ Fan (2012) |
|  | *Medicago sativa* L. | Leaf, stem, root | Bicoumarins | Dicoumarol | Yin ＆ Qin (2008) |
|  |  |  | Furanocoumarins | Alfalfa lactone, estrogenic lactone |  |
|  | *Psoralea corylifolia* Linn. | Decoction | Furanocoumarins | Psoralen, psoralidin | Zhang (2012) |
