# Supplementary material for: Anabolic metabolism of autotoxic substance coumarins in plants
Source: PeerJ. 2023 Dec 6;11:e16508. doi: 10.7717/peerj.16508 (PMC10710134; doi:10.7717/peerj.16508)
Supplement: Supplemental Information 8 [file peerj-11-16508-s008.docx]

**Table 3:**

**Distribution of plant coumarins**

| Plant species | Coumarins | Distribution characteristics of coumarins | | | Reference |
| --- | --- | --- | --- | --- | --- |
|  |  | Concentration position | Growth period | Stubbles |  |
| *Citrus grandis* (L.) Osbeck | Isoimperatorin, isomeranzin | Flavedo, albedo | Not reported | | Nie ＆ Zhao (2021) |
|  | Bergaptol, 6',7'-epoxybergamottin | Pulp |  |  |  |
| *Mikania glomerata* | Coumarin | Leaf, stem | young leaf > mature leaf | Not reported | de et al. (2007) |
| *Peucedanum praeruptorum* Dunn | Pyranocoumarins | Secretory canals | before bolting > after bolting | Not reported | Chen et al. (2019) |
| *Chrysanthemum segetum* L. | 7-methoxycoumarin | Root | Not reported | | Ochocka et al. (1995) |
|  | Angelica lactone | Aerial parts |  |  |  |
| *Angelica dahurica* | 37 species | Primary root (periderm, cortex, and phloem) | Not reported | | Gao ＆ Li (2023) |
|  | 36 species | Lateral roots (phloem) |  |  |  |
| *Levisticum officinale* W. D. J. Koch | Simple coumarins,  Pyranocoumarins | Root | Not reported | | Olennikov (2023) |
| *Heracleum sosnowski Mandena* | Coumarin | Seeds | Not reported | | Andreeva (2021) |
| *Notopterygium forbesii* Herb. Boiss. | Isoimperatorin | Root | Not reported | | Liu et al. (2012) |
| *Medicago sativa* L. | Coumarin | Leaf | podding stage > early flowering stage > bud stage > branching stage > seedling stage | third stubble > second stubble > first stubble | Rong (2017); Chon et al. (2016); Wang (2008); Wang et al. (2017) |
