# Supplementary material for: Anabolic metabolism of autotoxic substance coumarins in plants
Source: PeerJ. 2023 Dec 6;11:e16508. doi: 10.7717/peerj.16508 (PMC10710134; doi:10.7717/peerj.16508)
Supplement: Supplemental Information 9 [file peerj-11-16508-s009.docx]

**Table 4:**

**Difference of coumarins accumulation in different plant varieties under environmental stress**

| Plant species | Stress conditions | Differences in coumarin accumulation | Reference |
| --- | --- | --- | --- |
| *primitive and wild citrus species (Atalantia buxifolia* and *C. latipes)*  cultivated citrus species (*C. sinensis* and *C. reticulata*) | Pathogens, varieties | Primitive citrus and wild citrus synthesize phenolic compounds ( such as coumarin ) faster than cultivated citrus | Killiny ＆ Hijaz (2016); Zaynab et al. (2018); Hussain et al. (2019); Rao et al. (2021) |
| Ziyang Xiangcheng (*Citrus junosSieb*. ex *Tanaka*, CJ),  trifolicate orange (*Poncirus trifoliata* (L.) Raf., PT) | Iron deficiency, varieties | The expression of genes related to coumarin synthesis in Ziyang Xiangcheng was higher than that in trifoliate orange | Hui (2022) |
| two contrasting peanut (*Arachis hypogaea* L.) genotypes (GG7: fast growing and tall, TG26: slow growing and semi-dwarf) | Drought, varieties | The content of coumarin in GG7 genotype seedlings was significantly higher than that in TG26 genotype peanut seedlings. | Patel, Fatnani ＆ Parida (2021) |
